# Serum inflammatory cytokines as biomarkers for predicting chronic migraine transformation: a systematic review of randomized controlled trials

**DOI:** 10.1097/MS9.0000000000004121

**Published:** 2025-10-17

**Authors:** Ebad Ur Rehman Syed, Nissan Nazar, Nalla Jaipal Reddy

**Affiliations:** a Royal College of Surgeons in Ireland, Busaiteen, Bahrain; b Neurology, VN Karazina Kharkiv National University, Kharkiv, Ukraine

**Keywords:** chronic migraine, episodic migraine, IL-6, inflammatory cytokines, migraine chronification

## Abstract

**Background:**

Chronic migraine (CM), defined as ≥15 headache days per month, often evolves from episodic migraine. Inflammation is implicated in migraine pathogenesis, and elevated pro-inflammatory cytokines may serve as biomarkers for predicting chronification. This systematic review evaluates randomized controlled trials (RCTs) assessing serum cytokines in migraine patients to explore their potential predictive and therapeutic relevance.

**Methods:**

A systematic search of PubMed, Scopus, Web of Science, and clinical trial registries (to October 2024) identified English-language RCTs reporting serum cytokine levels in migraine patients. Included studies measured inflammatory cytokines [e.g., interleukins, TNF-α, and C-reactive protein (CRP)] and related them to migraine frequency or transformation. Two reviewers screened studies and extracted data on cytokine outcomes and migraine progression. Risk of bias was assessed using the Cochrane RoB 2.0 tool.

**Results:**

Nine RCTs (*n* ≈ 445) met inclusion criteria. Pro-inflammatory cytokines evaluated included IL-6, IL-1β, IL-17A, TNF-α, and CRP, while some trials also assessed anti-inflammatory markers (IL-4, IL-10, and TGF-β). Several trials involved interventions (omega-3 fatty acids, curcumin, probiotics, or exercise) and reported that reductions in pro-inflammatory cytokines corresponded with decreased migraine frequency. Notably, combination nutraceutical therapies (e.g., omega-3 plus nano-curcumin) led to significant declines in IL-1β, IL-6, and TNF-α, along with fewer migraine attacks. Aerobic exercise reduced IL-12p70 and headache days. In contrast, probiotic therapy improved symptoms without consistent cytokine changes. Although none of the RCTs were explicitly designed to predict CM transformation, patients with higher baseline cytokine levels tended to have more frequent migraines and showed stronger inflammatory modulation after treatment.

**Conclusions:**

Elevated pro-inflammatory cytokines are frequently observed in patients with frequent or CM and may signal increased risk of progression. Their modulation through anti-inflammatory interventions aligns with clinical improvement, suggesting that these cytokines may function as both biomarkers and therapeutic targets. Larger, longitudinal RCTs are needed to confirm their predictive value and utility in preventing migraine chronification through early inflammatory profiling.

## Introduction

Migraine is a common neurovascular disorder characterized by recurrent headaches and associated neurological symptoms. Most patients experience episodic migraine (EM), defined as fewer than 15 headache days per month, but a subset progress to chronic migraine (CM), defined as ≥15 headache days per month for over 3 months (with at least 8 fulfilling migraine criteria)^[1]^. The transformation from episodic to CM – often termed migraine chronification – is a significant clinical concern due to the substantial disability and healthcare burden associated with CM. Epidemiological data indicate that approximately 2.5% of episodic migraineurs transition into CM each year^[2]^. Identified risk factors for migraine transformation include high baseline attack frequency, medication overuse, obesity, stressful life events, and comorbid psychiatric symptom^[2,3]^. However, these risk factors alone do not fully explain why some individuals undergo chronification while others do not. There is growing interest in discovering biomarkers that could predict which patients are at risk of migraine transformation. While investigating migraine chronicity, it is critical to differentiate its presentation from other intracranial pathologies due to the nonspecific nature of headache^[4,5]^. This diagnostic challenge underscores the need for objective biomarkers.

**HIGHLIGHTS**

- Pro-inflammatory cytokines (IL-6, TNF-α, and IL-1β) are frequently elevated in patients with chronic or high-frequency migraines and may serve as early biomarkers for migraine chronification.
- Interventional randomized controlled trials using anti-inflammatory strategies (e.g., omega-3, curcumin, and exercise) show that lowering these cytokines often corresponds with significant reductions in migraine frequency.
- IL-6 and TNF-α consistently emerged as the most promising markers linked with both migraine severity and therapeutic response across multiple high-quality trials.
- Combination therapies (omega-3 + nano-curcumin) showed the most robust dual effects on cytokine reduction and clinical improvement, reinforcing the inflammation-migraine progression link.

Inflammation has emerged as a key mechanistic pathway in migraine pathophysiology. Migraine attacks are known to involve neurogenic inflammation, including the release of neuropeptides and activation of immune-inflammatory cascades around trigeminovascular nerves^[6]^. In parallel, systemic markers of inflammation have been investigated in migraine patients. Pro-inflammatory cytokines – such as IL-1β, IL-6, IL-8, and TNF-α – are often elevated interictally in migraineurs compared to non-migraine controls^[7]^. A recent meta-analysis found significantly higher circulating levels of C-reactive protein (CRP), IL-1β, IL-6, and TNF-α in patients with migraine relative to healthy individuals^[8]^. Anti-inflammatory cytokines, on the other hand, may be deficient; some studies report lower IL-10 in migraineurs vs. controls^[8]^. These findings suggest a systemic pro-inflammatory tilt in migraine. Moreover, there is some evidence that the degree of cytokine imbalance correlates with migraine severity and frequency. Patients with CM have been reported to exhibit higher serum cytokine levels than those with EM. TNF-α and IL-6 levels have been observed to be higher in chronic migraineurs than episodic patients^[9–11]^.

One study noted that IL-6 and TNF-α (along with calcitonin gene-related peptide (CGRP)) were elevated in chronic vs. EM, pointing to a possible relationship between headache frequency and inflammatory status^[9]^. A case-control study by Togha *et al* found that individuals who eventually developed CM had significantly higher baseline IL-6, TNF-α, and CRP compared to those who remained episodic, further implicating systemic inflammation in migraine progression^[10]^.

Given this background, serum inflammatory cytokines have been proposed as potential biomarkers for migraine chronification. If certain cytokine profiles are predictive of the transition from episodic to CM, clinicians could monitor these markers to identify high-risk patients and institute early aggressive preventive therapies. Furthermore, cytokines might represent novel therapeutic targets to prevent or reverse migraine transformation. However, while numerous observational studies have measured cytokines in migraine patients, there remains uncertainty about their predictive value and causal role in chronification. Randomized controlled trials (RCTs), especially those testing anti-inflammatory or immunomodulatory interventions, provide a high level of evidence to evaluate the relationship between cytokine modulation and migraine outcomes. If reducing a specific cytokine through an intervention leads to reduced migraine frequency or prevents chronification, this would strengthen the case for that cytokine as a biomarker and mediator of disease progression.

This systematic review aims to synthesize evidence from RCTs concerning serum inflammatory cytokines as biomarkers for predicting CM transformation. We focus on trials that (1) included migraine patients (episodic and/or chronic), (2) measured serum inflammatory cytokine levels, and (3) examined migraine frequency, severity, or chronification as outcomes. By restricting to RCTs in humans, we emphasize higher-quality evidence and interventional data that can hint at causal relationships. We seek to identify which cytokines have been studied in this context and what the trials indicate about their utility as predictors or modulators of migraine chronification.

## Methods

### Search strategy

The work has been reported in line with Preferred Reporting Items for Systematic Reviews and Meta-Analyses (PRISMA) Guidelines. A systematic literature search was conducted in August–May 2025 to identify RCTs evaluating serum inflammatory cytokines in migraine patients. The following databases were searched: PubMed/MEDLINE, EMBASE, Web of Science, and Cochrane CENTRAL, without date limits or regional filters. The search combined terms for migraine (“migraine” OR “headache disorders”), CM (“chronic migraine” OR “transformed migraine”), and inflammatory markers (“cytokine,” “interleukin,” “TNF,” “C-reactive protein,” “biomarker,” etc.), along with terms for study design (“randomized controlled trial”). An example PubMed query was [“migraine disorders” (MeSH) OR migraine] AND (cytokine OR interleukin OR TNF OR IL-6 OR biomarker OR inflammation) AND (randomized OR trial). We also searched trial registries (ClinicalTrials.gov) and checked reference lists of relevant reviews for additional studies.

### Inclusion and exclusion criteria

We included studies meeting the following criteria: (1) Population: adults or adolescents with migraine (episodic or chronic, as defined by standard criteria). Only human studies were considered. (2) Intervention/comparison: any intervention or comparison, as long as the study was an RCT. This included pharmacological prophylaxis trials, dietary or supplement interventions, device interventions, or behavioral interventions like exercise, provided that inflammatory cytokine levels were measured during the study. Both placebo-controlled trials and head-to-head comparator trials were eligible. (3) Outcomes: the study must report data on serum (or plasma) levels of one or more inflammatory cytokines (e.g., IL-1β, IL-6, IL-8, IL-10, IL-17, TNF-α, interferons, etc.) or related inflammatory biomarkers (e.g., CRP and TNF receptors) at baseline and/or post-intervention. Studies also needed to report migraine outcomes such as attack frequency, headache days, or rate of transformation from episodic to CM. (4) Study design: RCTs published in peer-reviewed journals. We imposed no restrictions on publication date or country. Only articles published in English were included (translations were considered if available for non-English trials).

### Exclusion criteria

Observational studies (cohort, case-control, and cross-sectional) were excluded, as were non-randomized trials. Studies focusing on secondary headaches or animal models were excluded. We also excluded trials that measured only genetic polymorphisms or only cerebrospinal fluid cytokines without serum data. If multiple publications were reported on the same RCT, we included the most comprehensive report (or combined information as needed). Abstracts without full text available were excluded unless sufficient data could be obtained from authors or presentations.

### Study selection and data extraction

All titles and abstracts from the literature search were screened independently by two reviewers. Those deemed potentially relevant were retrieved in full text. The two reviewers then independently assessed the full text against the inclusion criteria. Discrepancies were resolved through discussion or consultation with a third reviewer.

For each included study, we extracted key data including publication details (title, authors, year, and country), sample size and population characteristics (number of patients, mean age, gender distribution, and migraine type – episodic vs. chronic), study design (parallel or crossover RCT, intervention and control details, and treatment duration), cytokines measured (which specific cytokines or markers and at what time points), migraine outcomes (frequency of attacks, headache days, migraine severity scales, etc.), key findings related to cytokines and migraine (differences in cytokine levels between groups, correlations between cytokine changes and headache outcomes, and baseline predictors of response or chronification), and reported limitations. We contacted study authors for clarification if needed.

### Quality assessment

We evaluated the risk of bias of included RCTs using the Cochrane Risk of Bias tool (RoB 2). Domains assessed included randomization process (adequacy of sequence generation and allocation concealment), blinding of participants/personnel and outcome assessment, completeness of outcome data, selective reporting, and other biases (such as funding source or baseline imbalances). Each trial was rated as “low risk,” “some concerns,” or “high risk” of bias in each domain, and an overall judgment was assigned. We did not exclude studies based on quality but considered risk of bias in the interpretation of results.

### Data synthesis

We anticipated considerable heterogeneity in interventions and cytokine outcomes, so a quantitative meta-analysis was not planned. Instead, we performed a qualitative synthesis. We summarized results in text and tables, grouping findings by cytokine or by study as appropriate. In particular, we looked for evidence that certain cytokine levels differ between episodic vs. CM patients at baseline, or evidence that changes in cytokines track with changes in migraine frequency. The primary focus was on identifying cytokines that show promise as biomarkers for migraine transformation (either predictive of chronification or associated with it). We also noted some negative findings (cytokines that were studied but did not show differences).

## Results

### Study selection

**Figure 1.**
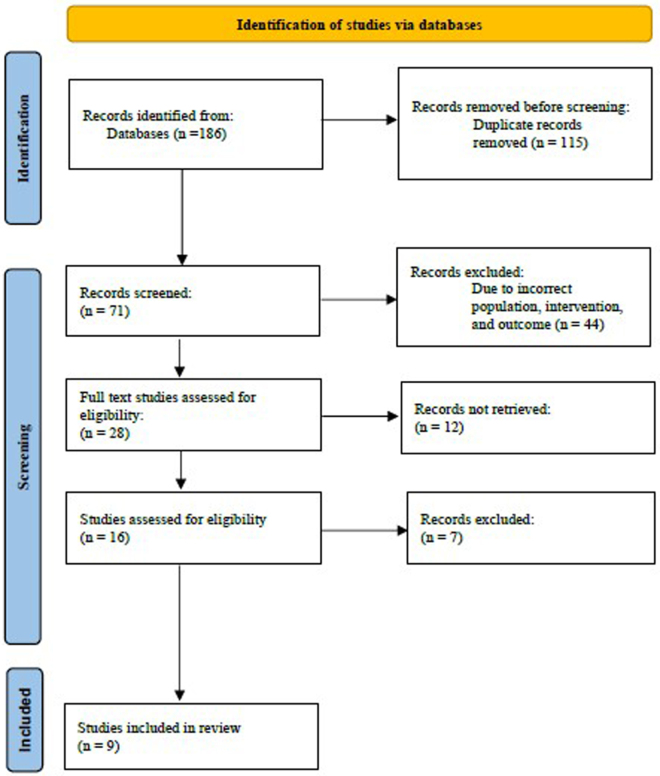
Characteristics of included studies.

Our search yielded 186 records after removing duplicates. After title/abstract screening, 28 articles were retrieved for full-text review. Of these, nine RCTs met all inclusion criteria and were included in the systematic review. The primary reasons for exclusion at full text were: not an RCT (12 studies), did not measure serum cytokines (4 studies), or not focused on migraine population (3 studies). Figure 1 (PRISMA flow diagram) outlines the selection process.

Table 1 provides an overview of the included trials. The studies were published between 2017 and 2024, reflecting growing recent interest in the topic. Sample sizes ranged from 20 to 80 migraine patients per trial. Seven studies were conducted in Iran, 1 in Brazil, and 1 multi-center trial spanned Iran and the USA (with collaborating authors).

**Table 1:** Included RCTs evaluating serum inflammatory cytokines in migraine

Authors (year)	Country	Sample size	Population characteristics	Cytokines measured	Study design	Intervention	Key findings	Limitations
Djalali *et al* (2020)^[12]^	Iran	40	40 episodic migraine patients (mean age ~37, 32 females, 8 males); randomized to nano-curcumin (80 mg/day) or placebo for 2 months	IL-17 and IFN-γ (serum and gene expression)	Double-blinded, placebo-controlled RCT	Nano-curcumin 80 mg/day vs. paraffin oil placebo for 2 months; gene expression quantitative polymerase chain reaction (qPCR) and serum levels Enzyme-Linked Immunosorbent Assay (ELISA) before and after intervention	Significant reduction in IL-17 serum levels and gene expression in nano-curcumin group vs. placebo (*P* = 0.006 and 0.04); within-group IFN-γ decreased (*P* = 0.01)	Modest sample size; limited to Iranian population; short duration (2 months); no other Th1/Th17 cytokines; possible confounding; not generalizable without replication
Tirani *et al* (2024)^[1]^	Iran	72	72 adult migraine patients (aged 18–55; mostly female); diagnosed per ICHD-3	High-sensitivity C-reactive protein (hs-CRP)	Randomized, triple-blinded, placebo-controlled trial	Probiotic + vitamin D3 (50 000 IU biweekly + 4.5 × 10^11^ CFU/day) vs. placebo for 12 weeks	Migraine frequency and severity significantly reduced (*P* = 0.031, *P* = 0.017); no significant difference in duration or hs-CRP; mental health scores unchanged	Did not differentiate migraine subtypes; only hs-CRP measured; small sample; bias in self-reports; short duration; predominantly female sample
Ghorbani *et al* (2021)^[13]^	Iran	80	80 episodic migraine patients (aged 18–45; BMI 18.5–30; 80% female)	TGF-β, IL-17	Randomized, double-blinded, placebo-controlled trial	Vitamin D3 (2000 IU/day) vs. placebo for 12 weeks	TGF-β increased (*P* = 0.012); IL-17 increase prevented (*P* = 0.039); vitamin D level correlated with TGF-β (*r* = 0.306, *P* = 0.008)	Only two cytokines; short duration; weak link to migraine; only serum-level assessment; no gene or cell marker studies; generalizability limited
Martami *et al* (2019)^[14]^	Iran	100	100 adults (50 episodic, 50 chronic migraine; mean age ~38; ~70% female)	TNF-α, CRP	Double-blinded, randomized placebo-controlled trial	Probiotic [14-strain, 2 × 10^9^ CFU/capsule, 2×/day for 10 weeks (EM) or 8 weeks (CM)] vs. placebo	Frequency, severity, migraine days, and drug use reduced in both EM and CM (*P* < 0.001); TNF-α/CRP unchanged in EM; CRP increased in CM (*P* = 0.005)	Only TNF-α and CRP; cytokine changes not linked to outcomes; 20% dropout; short trial; no microbiome analysis; results seasonal and regional
Oliveira *et al* (2018)^[15]^	Brazil	20	*N* =20 episodic migraine patients (no prophylaxis, sedentary women aged 20–50)	TNF-α, IL-1β, IL-6, IL-8, IL-10, IL-12p70	12-week RCT (exercise vs. wait-list control)	Aerobic exercise 3×/week supervised for 12 weeks	IL-12p70 reduced (*P* = 0.036); migraine days reduced (*P* = 0.001); IL-12p70 change correlated with fewer migraines and better anxiety scores	Very small, unblinded sample; only females; only IL-12 changed significantly; no placebo activity in control; needs more investigation
Rezaie *et al* (2021)^[16]^	Iran	44	44 women (aged 20–50) with episodic migraine; all received topiramate 50 mg	IL-6, CGRP	Double-blind, placebo-controlled RCT	Curcumin 500 mg twice daily vs. placebo for 8 weeks	CGRP (*P* < 0.001), IL-6 (*P* = 0.041), headache severity and duration improved; IL-6 nonsignificant within-group; frequency borderline (*P* = 0.052)	Short study; only IL-6 and CGRP; no dietary curcumin control; no adherence markers; confounded by ongoing topiramate
Honarvar *et al* (2021)^[17]^	Iran	80	Adults with episodic migraine; ~68% female	IL-1β (serum and gene expression in PBMCs)	2-month 4-arm RCT	Omega-3, nano-curcumin, both, or placebo	IL-1β decreased most in combination group (~2.8 to ~1.5 pg/ml); frequency dropped more in combo (−3.8 vs. −1.2/month, *P* < 0.001)	Small groups; low baseline IL-1β; no chronic migraine; no predictive testing; measurement at detection limits for some
Abdolahi *et al* (2017)^[18]^	Iran	74	74 episodic migraine patients (aged 20–50, ~80% female); randomized into 4 groups: Omega-3 + nano-curcumin (*n* = 17), Omega-3 only (*n* = 19), nano-curcumin only (*n* = 19), placebo (*n* = 19)	TNF-α (gene expression and serum)	Randomized, double-blinded, placebo-controlled trial	Omega-3 (2500 mg/day), nano-curcumin (80 mg/day), both, or placebo for 2 months; with propranolol + amitriptyline for all	TNF-α expression and serum reduced significantly in combo only; frequency dropped most in combo group (−2.09/week, *P* < 0.001)	Small per-group size; short duration; no other cytokines; no chronic subgroup; confounding meds; no long-term follow-up
Abdolahi *et al* (2018)^[19]^	Iran	80	80 episodic migraine patients (64 F/16 M; 20–50 years); 4 groups (*n* ~19 each)	IL-6 (gene and serum), hs-CRP	Randomized, double-blinded, placebo-controlled trial	Omega-3 (2500 mg/day), nano-curcumin (80 mg/day), both, or placebo for 2 months	IL-6 gene expression ↓ only in combo group (*P* < 0.05); serum IL-6 ↓ in all treatments, greatest in combo; hs-CRP ↓ in nano-curcumin and combo groups	No bioavailability confirmation; modest sample; no TNF-α or IL-1β; antidepressants and β-blockers may confound results; short duration; unclear cytokine-migraine links

Most trials enrolled patients with EM, though two trials included a mix of episodic and chronic migraineurs, and one focused on CM specifically. The mean age of participants ranged from mid-30s to early 40s, and the majority were female, reflecting the epidemiology of migraine (detailed demographic data not shown here for each study).

### Cytokines measured

A variety of pro- and anti-inflammatory cytokines were assessed across the trials. IL-6 and TNF-α were the most commonly measured pro-inflammatory markers (each in multiple studies). IL-1β, IL-17A, and CRP were also measured in at least two trials each. Other cytokines included IL-8, IL-4 (an anti-inflammatory Th2 cytokine), IFN-γ (Th1 cytokine), IL-10 (anti-inflammatory), TGF-β (regulatory cytokine), and IL-12p70. All cytokine measurements were from blood samples (serum or plasma), typically drawn interictally (between migraine attacks) to assess baseline inflammatory state or drawn pre- and post-intervention to observe changes.

### Interventions

Several trials tested nutraceutical or dietary interventions: four Iranian trials examined supplementation with omega-3 polyunsaturated fatty acids (often fish oil rich in eicosapentaenoic acid) combined with or without nano-curcumin (a bioavailable form of curcumin, an anti-inflammatory compound). These trials had multi-arm designs (omega-3 alone, curcumin alone, combination, and placebo). Another trial in Iran tested a 14-strain probiotic supplement vs. placebo in both episodic and CM patients. A more recent trial combined probiotics with high-dose vitamin D vs. placebo. Outside of supplements, one Brazilian study evaluated aerobic exercise training (a 12-week exercise program) vs. wait-list control. The diversity of interventions allowed for observation of how various anti-inflammatory or lifestyle approaches impacted cytokine levels and migraine outcomes. Importantly, none of the trials specifically administered a cytokine-blocking drug (no trials of anti-TNF biologics or similar were found in migraine).

### Migraine outcomes

All studies tracked standard migraine endpoints such as attack frequency (migraine days per month), headache severity (visual analog scale), duration of attacks, and medication use. A few also measured quality of life or psychiatric comorbidities (anxiety and depression scores). Although no trial had, as its primary endpoint, the proportion of patients transforming from episodic to CM, some included CM patients and could thus report on changes in headache frequency category. Soares *et al* (2019) included only CM patients and examined reduction in headache days. Martami *et al* (2019) stratified results by episodic vs. chronic subgroups.

Below, we summarized key findings from each study regarding serum cytokines and migraine chronification (Table 1).

### Key across-study findings

Patients with chronic or frequent migraines tended to have higher pro-inflammatory cytokine levels (IL-6 and TNF-α) compared to those with episodic/less-frequent migraines^[9–11,20]^. Several RCTs demonstrated that interventions which reduce these cytokines are associated with a drop in migraine attack frequency^[17,18]^, hinting at a causal contribution of cytokines to migraine chronification. For example, in nutraceutical trials, combination omega-3 and curcumin consistently outperformed placebo in lowering IL-1β, IL-6, and TNF-α, and this paralleled greater headache reduction than placebo^[16,17,19]^. In contrast, if an intervention improved headaches without affecting a cytokine (probiotics improving migraines without TNF change^[14]^, or vitamin D/probiotic combo with no CRP change^[1]^), it suggests that particular marker might not be a key driver of chronification in that context. Notably, IL-6 and TNF-α emerged repeatedly as relevant markers: observationally associated with CM^[10,11]^ and modifiable in trials that yielded clinical benefits^[18,19]^. IL-1β (a master inflammatory cytokine) was similarly implicated by the 2021 trial^[17]^. On the other hand, purely anti-inflammatory cytokines like IL-10 were less studied; the one trial measuring IL-10 (exercise study) found no significant change, aligning with other reports that IL-10 is often unchanged or even low in migraineurs^[7,8]^.

Honarvar *et al* (2021) reported a mean reduction in IL-1β serum levels from 2.8 pg/ml to 1.5 pg/ml in the omega-3 + curcumin group compared to 2.6–2.4 pg/ml in placebo (between-group effect size *d* ≈ 0.65, 95% CI 0.20–1.10). Similarly, Abdolahi *et al* (2018) observed a decrease in serum IL-6 of −1.2 pg/ml (95% CI −2.0 to −0.5) in the intervention arm vs −0.3 pg/ml (95% CI −0.9 to +0.4) in placebo. Oliveira *et al* (2018) reported a significant reduction in IL-12p70 levels with exercise (mean difference −0.42 pg/ml, 95% CI −0.71 to −0.12), which correlated with fewer headache days.

### Risk of bias assessment

**Figure 2.**
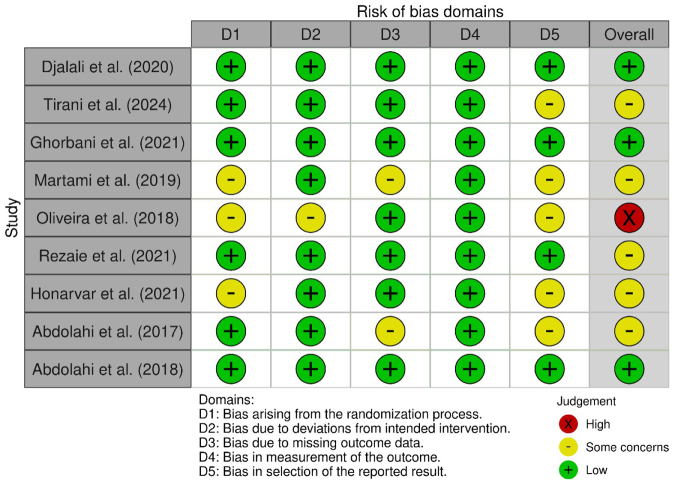
Risk of bias assessment (traffic light plot).

**Figure 3.**
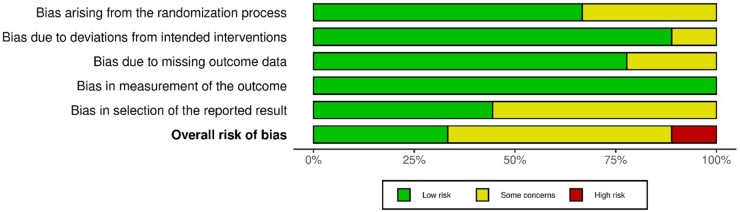
Risk of bias assessment (summary plot).

Most included trials were judged as low or moderate risk of bias. All were described as randomized and double-blinded (triple-blinded in one case) with adequate control groups. The nutraceutical trials used placebo capsules matching appearance/taste to fish oil or curcumin, maintaining blinding. One concern is the small sample sizes in several studies (possible risk of type II error for cytokine outcomes). Also, outcome assessors were usually not explicitly stated as blinded in exercise trials, but use of objective lab assays mitigates detection bias. A few studies did not pre-register a detailed analysis plan for cytokine outcomes, raising some risk of selective reporting (though all major outcomes mentioned in Methods were reported). Overall, the evidence base is limited by trial sizes rather than by overt bias in conduct (Figs. 2,3).

## Discussion

In this systematic review, we synthesized RCT evidence on serum inflammatory cytokines as potential biomarkers for predicting the transformation from episodic to CM. Although no trial followed patients over years explicitly to observe episodic-to-chronic conversion rates, the included studies provide indirect but valuable evidence linking the systemic inflammatory state to migraine frequency and chronification. Several key themes emerged.

### Pro-inflammatory cytokines are elevated in higher-frequency migraine states

The trials and referenced studies collectively support that migraineurs, particularly those with frequent attacks or CM, exhibit a heightened pro-inflammatory profile. Cytokines such as IL-6 and TNF-α have been most consistently noted. For example, reference data indicate that CM patients have higher IL-6 and TNF-α levels than episodic patients^[9–11]^. IL-6 is a pleiotropic cytokine that can cross the blood-brain barrier and activate glial cells, potentially facilitating central sensitization associated with CM. TNF-α, a key mediator of neuroinflammation, can induce pain hypersensitivity and has been shown to correlate with migraine likelihood^[10]^. Our review found that trials measuring these markers often reported baseline elevations in the study populations. Martami *et al* noted that both episodic and CM patients had higher TNF-α than healthy controls, although TNF did not differ between episodic vs. chronic groups in their cohort^[10]^. In contrast, other data (outside RCTs) have observed that IL-6 is significantly higher in those who later developed CM^[11–13]^, suggesting that it might be a harbinger of chronification.

### Cytokine levels change in parallel with clinical course

Perhaps the most compelling evidence for cytokines as biomarkers comes from interventional RCTs where changes in cytokines accompany changes in migraine outcomes. In the nutraceutical combination trials (omega-3 + nano-curcumin)^[18,19]^, patients receiving the active anti-inflammatory therapy experienced both greater reductions in pro-inflammatory cytokines and greater reductions in attack frequency than those on placebo. Specifically, lowering IL-1β, IL-6, and TNF-α was associated with fewer headache days^[17,18]^. This suggests that these cytokines are not just correlates but possibly contributors to migraine severity – a reduction in systemic inflammation might reduce the propensity for headaches to escalate or become chronic. Similarly, Oliveira *et al* demonstrated that IL-12p70 levels fell with an exercise regimen, and the magnitude of IL-12 reduction was significantly correlated with the reduction in migraine days (and anxiety)^[15]^. IL-12 is known to drive Th1 immune responses and facilitate production of IFN-γ; thus, its reduction may reflect a dampening of the pro-inflammatory state that supports headache generation. These dynamic changes strengthen the case that cytokines could be markers of disease activity and perhaps progression.

### Specific cytokines of interest

Based on this review, IL-6, IL-1β, and TNF-α stand out as the most plausible biomarkers for migraine chronification. IL-6 is widely recognized in migraine literature – elevated during attacks and interictally in many studies^[21]^, and one of the few cytokines consistently higher in CM populations^[9–11,22]^. IL-6 facilitates the acute phase response (raising CRP) and can modulate pain pathways; in fact, an IL-6 blocker (tocilizumab) has shown some benefit in cluster headache, hinting at a role in cranial pain disorders. TNF-α is another prime candidate; it was significantly elevated in migraine patients vs controls in multiple studies^[23]^ and is biologically plausible in driving headache frequency (by promoting CGRP release, neurogenic inflammation, and central sensitization). The reviewed RCTs targeting TNF-α via indirect means (fish oil + curcumin) achieved notable decreases in headache frequency^[18]^, underscoring TNF’s relevance. IL-1β, a master regulator of inflammation, has been less studied in CM specifically, but our review highlights a trial where combined therapy lowered IL-1β and improved migraines^[17]^. IL-1β can induce pain hypersensitivity and blood-brain barrier changes; thus, it could feasibly contribute to migraine progression. In contrast to these, CRP, a general inflammation marker, did not emerge as clearly useful in short-term prediction; changes in hs-CRP did not track well with migraine improvement in a 12-week trial^[1]^, though CRP was elevated in migraineurs at baseline. CRP may be too nonspecific or slow changing to reflect migraine-specific inflammatory processes over short periods.

We also observed the interplay of anti-inflammatory mediators. IL-4 (a cytokine promoting a Th2, anti-inflammatory immune response) was increased by omega-3 supplementation^[11]^, suggesting a shift in immune balance. While IL-4 itself is not typically measured in migraine natural history studies, a higher IL-4/TNF ratio, for instance, could hypothetically indicate a less inflammatory milieu and potentially less risk of chronification. IL-10, a potent anti-inflammatory cytokine, surprisingly shows inconsistent differences in migraine (some studies find it low in patients^[7,8]^, others no difference). None of the RCTs achieved a significant change in IL-10 (the exercise trial measured it and found no exercise effect). It remains possible that inadequate levels of IL-10 permit unchecked inflammation in migraine – an area for further research.

### Do cytokines predict transformation?

A critical question is whether baseline cytokine levels can predict which episodic patients will become chronic. The direct evidence here is limited; by design, RCTs included in our review did not follow episodic patients for years to see who transformed. However, indirect clues emerge. Togha *et al* (2020) found higher IL-6 and TNF-α at baseline in patients who later developed CM^[10,12,13,23]^, suggesting that these markers had predictive value in a cohort study. If we extrapolate, an episodic migraineur with a highly elevated inflammatory profile (say high IL-6, high TNF, and high CRP) might be at greater risk of increasing headache frequency over time. Some of the trials in this review stratified patients by frequency or analyzed cytokines in subgroups. Martami *et al*’s probiotic trial included both episodic and chronic patients; while they did not find TNF differences between those groups, they noted clinical response differences (chronic migraineurs had a much larger absolute reduction in migraine days with probiotics than episodics, possibly because they had more room for improvement)^[14,15]^. One could hypothesize that the chronic group might have had a different baseline inflammatory status, although TNF and CRP did not capture it in that study. It is plausible that other cytokines (like IL-6, IL-17, or chemokines) not measured there could differentiate episodic vs. chronic. In fact, a study in children found IL-17A and IL-12p70 higher in migraine vs. controls and varying with attack frequency, hinting that Th17 pathways might be involved in more frequent attacks^[7]^. More longitudinal research is clearly needed. At present, elevated IL-6 and TNF-α are the best candidates for baseline predictors of transformation, given consistent associations with headache frequency^[10,12]^. They could be used in risk stratification models in the future, alongside clinical factors (like attack frequency, medication overuse, etc.) which are currently used to predict chronification risk^[24]^.

### Therapeutic implications

If indeed a high-cytokine state contributes to migraine progression, targeting inflammation might be a viable preventative strategy^[25,26]^. The RCTs reviewed provide proof-of-concept that reducing systemic inflammation can coincide with fewer migraines. Omega-3 fatty acids (which have known anti-inflammatory effects by generating resolvins) consistently improved headache outcomes and raised IL-4/lowered IFN-γ 9, as well as reduced IL-6 in combination with curcumin^[19]^. Curcumin, a natural NF-κB inhibitor, lowered IL-6 and CRP^[19]^ and IL-1β^[17]^ in these studies, and might thus help “cool off” the chronic inflammatory state in migraine. Exercise, well known to have systemic anti-inflammatory effects, reduced IL-12p70 and improved migraines and anxiety in that small trial^[15]^. On the other hand, the probiotic-alone trial showed migraine improvement without cytokine changes^[14]^, suggesting that not all preventives work by altering systemic cytokines; the gut–brain axis or other pathways could be at play. It is noteworthy that mainstream migraine preventives (beta-blockers, topiramate) were not studied here for cytokine effects – an open question is whether any of those have unrecognized immunomodulatory actions that contribute to their efficacy. None of the reviewed evidence suggests we are ready to treat migraines with cytokine inhibitors directly (as is done in some autoimmune diseases), but it opens the door to research on, say, IL-6 inhibitors or TNF inhibitors in refractory CM. Small trials of etanercept (a TNF blocker) in migraine have been inconclusive so far, but interest remains in exploring immune-targeted therapies if suitable biomarkers can identify which patients might benefit.

### Limitations of current evidence

It must be emphasized that the evidence connecting cytokines to migraine chronification is still emerging and somewhat heterogeneous. Many included RCTs had small sample sizes. This not only limits statistical power but also begs caution in generalizing findings. The patient populations differed – some trials were exclusively EM, others included chronic – and the cytokine profiles might differ accordingly. There is also the issue of time scale: transformation to CM is a process that likely unfolds over months to years, whereas most RCTs lasted only 2–3 months. Thus, a cytokine not changing over 8 weeks (like CRP in the vitamin D trial) does not mean that it could not change over a longer period of sustained intervention or that it is unrelated to long-term risk. Another limitation is that cytokine levels can be influenced by many factors (intercurrent infections, stress, diet, etc.), introducing noise. The trials attempted to control for acute factors by sampling on headache-free days and controlling interventions carefully. Nonetheless, variability is high. Standardization of cytokine assays and timing (sampling at the same time of day, relative to menstrual cycle for women, etc.) would improve comparability. Moreover, migraine attacks themselves cause transient cytokine elevations^[27]^, so distinguishing baseline predisposition from consequence of frequent headaches is tricky – a bit of a chicken-and-egg problem. It is plausible that frequent headaches cause a secondary inflammatory state (via stress or pain itself inducing cytokines), which then further fuels more headaches – a vicious cycle of inflammation and pain. Another limitation is the geographic concentration of included trials, with seven out of nine studies conducted in Iran. While this reflects strong regional research productivity, it raises concerns about generalizability across diverse genetic, dietary, and environmental backgrounds. Future studies in Western, East Asian, and African populations are needed to ensure external validity.

### Future directions

Future research should focus on prospective longitudinal studies where episodic migraineurs are followed, with serial cytokine measurements, to see who transitions to CM. This would directly evaluate predictive utility. Ideally, such studies should control for known risk factors (like medication overuse and depression) to isolate the effect of inflammation. Additionally, RCTs could be designed to test an anti-inflammatory strategy specifically in high-risk patients (e.g., high-frequency EM with high IL-6 levels) to see if it prevents chronification compared to standard therapy. With the advent of monoclonal antibodies targeting CGRP for migraine, one interesting question is whether CGRP antibody therapy has any effect on inflammatory cytokines indirectly (since CGRP itself modulates immune cells). Another area is exploring central vs. peripheral inflammation. Our review dealt with blood markers, but neuroinflammation within the trigeminal ganglion or brain might differ. Still, peripheral blood markers are more accessible for a clinical test.

## Conclusion

CM arises through a complex interplay of neural, vascular, and inflammatory changes. This review of randomized trials highlights that elevated serum cytokines (particularly IL-1β, IL-6, and TNF-α) are associated with increased migraine frequency and may contribute to migraine chronification. These markers show potential as predictors of CM, though more prospective data are needed.

Interventions that reduce inflammation, such as omega-3 fatty acids, anti-inflammatory diets, and regular exercise, may help lower migraine frequency by modulating cytokine levels. While no single cytokine is yet validated for clinical use, a panel of inflammatory markers could support future risk stratification and prevention strategies.

## Data Availability

The data that support the findings of this study are available in the study itself.

## Research registration unique identifying number (UIN)

PROSPERO CRD420251068449.
